# Emerging Infectious Agents and Blood Safety in Latin America

**DOI:** 10.3389/fmed.2018.00071

**Published:** 2018-03-14

**Authors:** José Eduardo Levi

**Affiliations:** ^1^Hospital Israelita Albert Einstein, São Paulo, Brazil

**Keywords:** blood transfusion, arboviruses, Latin America, Zika, Dengue, Chikungunya

## Abstract

Historically, emerging infectious agents have been an important driving force toward the enhancement of blood safety, illustrated by the sharp reduction in the transmission of infectious agents by blood transfusion after human immunodeficiency virus (HIV) epidemics. In general, Latin American (LATAM) countries have introduced screening for microorganisms with proven blood transmission with some delay in comparison to developed countries, but, nowadays, all LATAM countries comply with a minimum standard of screening which includes Hepatitis B, C, HIV, *Treponema pallidum*, and *Trypanosoma cruzi*. Noticeably, all those agents, in addition to HTLV, cause chronic infections. By contrast, in the last decade, the region has witnessed explosive outbreaks of arboviral diseases, representing a new challenge to the blood system, threatening not only blood safety but also availability. So far, the clinical impact of transfusion-transmitted Dengue, Chikungunya, or Zika has not been evident, precluding immediate reaction from the authorities. A number of other arboviruses are endemic in the region and may, unpredictably, originate new epidemics. Several measures must be taken in preparedness for the potential emergence of another arbodisease.

## Introduction

The first agent verified to be transmitted by blood transfusion was the malaria protozoa (1) followed by Syphilis, the latter leading to the introduction of predonation testing in the first decades of the previous century (2). During the next 70 years, the safety of blood transfusions was gradually increased, covering a growing range of agents, more importantly the hepatitis viruses and human immunodeficiency virus (HIV). In common, all these pathologies have a short symptomatic acute phase and a variable rate of progression to a chronic asymptomatic period that may last through life. Blood units collected from unaware infected donors are averted from being transfused by detecting specific antibodies against those agents. With the exception of the hepatitis B virus (HBV) surface antigen (HBsAg) detection, the hallmark of prevention by laboratorial analysis has been the development and implementation of imunoassays, including, in some countries, the anti-hepatitis B core antigen (anti-HBc), that detects occult B carriers lacking, by definition, detectable HBsAg.

By the beginning of the millennia, the evolution of nucleic acid testing (NAT) allowed their incorporation to the blood screening routine, in order to interdict window-period donations, pursuing for an unattainable zero risk. A better selection of candidate donors with more stringent epidemiological and behavioral restrictions, summed to the arsenal of screening tests, have dropped the risk of transfusion transmission of infectious agents to a very low level, being nowadays a rare event (3).

Latin American (LATAM) countries are in general still struggling to motivate the population to donate blood voluntarily and regularly. Donations in the region are commonly insufficient for sustaining the transfusion demand, thereby resulting in a permanent blood shortage. In many countries, most of the donations come from replacement, often familiar donors (4) and emphasis is given to transform those into altruistic regular donors. Surprisingly, it has been observed that in some centers, repeat donors pose a risk that is similar or even higher than first-time donors concerning HIV transmission (5). This observation is justified by a fraction of repeat donors being indeed composed of HIV test seekers (6).

With some delay in comparison to developed countries, the four most important screening targets, HBV, HCV, HIV, and Syphillis, in addition to *Trypanosoma cruzi*, of local uttermost importance, were fully implemented in the 1990s (4). Seroprevalence of these agents were and are still much higher when compared to those verified in blood donors from Europe, Japan, Canada, and the US (3, 4, 7).

In this scenario, the explosive outbreaks of arboviruses with a high rate of asymptomatic subjects with a short-term viremia is new to the LATAM blood transfusion community; thus, there is a lot of uncertainty in how to deal with it. There are several proposed measures to mitigate this situation: averting blood collection in affected regions, applying pathogen reduction methods for plasma and platelet concentrates, adopting NAT and quarantine while waiting for post-donation information of donor’s health. Some countries may implement all them and others one or none. Certainly, this variability is not only due to scientific gaps in our knowledge but also to the resources available and political determination in each country or region. Table 1 summarizes selected features of the arboviruses representing today potential threats to the blood supply in LATAM.

**Table 1:** Selected features of emerging viruses representing a potential threat to the blood supply in Latin America, 2018.

	Arbovirus		
	Chikungunya	Dengue	Zika
Family/genus	Togaviridae/alphavirus	Flaviviridae/flavivirus	Flaviviridae/flavivirus
Enveloped	Yes	Yes	Yes
Viremic blood donors	Yes	Yes	Yes
Proven TT	No	Yes	Yes
NAT screening commercially available	No	No	Yes
Inactivated by PIT^a^	Yes	Yes	Yes
Vaccine available	No	Yes	No

*^a^Pathogen reduction/inactivation technologies*.

## Emerging Viruses

### Dengue

Viruses transmitted by arthropods (arboviruses) have always been of concern to human health but never much in the radar of blood banks. Dengue was the first arbovirus to cause epidemics in a global scale in the twenty-first century, globally affecting millions, from the Far East to the Americas. In LATAM, Brazil has been the country with the largest number of cases, experiencing yearly outbreaks from moderate to high intensity. Moreover, all four serotypes are now endemic, but there is still a large number of subjects naïve to at least one of the four serotypes, meaning that outbreaks will continue to occur.

The main impact of dengue outbreaks to the blood system is the fall in the number of candidate donors, thus shortening the supply of blood products, aggravated by the universal practice of transfusing dengue patients with low platelet counts (8). As most dengue infections are asymptomatic, it is likely that such infected subjects are be able to donate and thus eventually transmit the dengue virus to recipients. This possibility was demonstrated by several studies detecting viremic donors in Brazil (9–11), Honduras (9), Mexico (12), and Puerto Rico (13) among others.

However, there is an obvious discrepancy in between the dengue incidence and rates of viremia verified among blood donors during outbreaks and the paucity of reports of dengue cases by the transfusional route (TT-DENV). The most comprehensive study on TT-DENV showed that recipients have an approximately 36% risk to get the virus from a viremic donor, but was unable to depict dengue-specific symptoms on those infected (11). Several reasons have been presented to explain this (un)finding, discussed in detail elsewhere (14), but may be summarized as follows: it seems that dengue viruses are well adapted to the mosquito to human cycle, and, passage through the invertebrate host and inoculation by its bite are required to cause disease on us. So, although TT-DENV is a recognized risk in many endemic countries in LATAM, preventive measures were never taken, since it did not convince clinicians and authorities of its morbidity for recipients. However, it is necessary to emphasize that, in rare instances, severe dengue-associated symptoms were observed on recipients of viremic donations (13, 15), thereby leading to the implementation of laboratorial screening in Puerto Rico, first by using NS1 antigen testing further replaced by NAT (16) but in nowhere else in LATAM.

### West Nile Virus (WNV)

The diverse outcome of the different Flaviviruses causing human diseases requires that each viral species to be studied in depth, making analogies and generic Flavivirus models of little practical utility. This was well illustrated when the WNV arrived to the US by the end of the previous century. It took about 2 years to get enough evidence of its aggressiveness when acquired by blood transfusion or organ transplantation (17), since there was no previous recognition of any arbovirus transmission by these modes. When this link was unquestionably proven, it triggered a fast response from the transfusion medicine community, culminating in the introduction of screening by NAT (18). As WNV moved so fast from East to West US, it seemed inevitable that it would spread further South to LATAM, since susceptible vectors are abundant in the region and there is a huge migration of bird species that may harbor high titer viremias, from US and Canada to South America. Contrary to expectations, WNV was never able to cause human outbreaks in LATAM (19).

### Zika

The trajectory of Zika virus (ZKV) from an obscure agent to a global health emergence has been comprehensively described (20). The well-studied outbreak in French Polynesia revealed the important association of ZKV to Guillain-Barré syndrome (21), while Brazil was the country to raise and prove the hypothesis of a shocking causal association of this Flavivirus to microcephaly and other fetal neural abnormalities (22). Concerns about blood safety were raised by Musso and co-workers in French Polynesia where NAT, pathogen inactivation and quarantine were deployed to protect the blood supply (23). So far, there are only two published clusters of TT-ZKV, both from Brazil (24, 25). Similar to TT-DENV, on those reports it was shown that ZKV was indeed transmitted to transfusion recipients but they did not develop any symptom associated with Zika disease. In French Polynesia, look-back of 12 recipients transfused with red blood cells from ZKV-RNA^+^ donors has not also identified any post-transfusion symptoms (23). Even though solid evidence for a severe clinical outcome of TT-ZKV is still missing, the precautionary principle led Fundação Pró-Sangue/Hemocentro de São Paulo, Brazil, to develop a validated in-house NAT (26) and adopt it, from February 2016 on, to provide Zika-RNA-free blood units to approximately 20 pregnant women per month. In Martinique, Guadaloupe, and the French Guyana, pregnant women received blood collected in mainland France (Xavier de Lamballerie, personal communication) while donations were screened by individual NAT in Marseille, France (27). This policy of prioritizing groups at higher risk such as pregnant and highly transfused fertile women, and fetuses was further advocated by experts and organizations in the field (28) and WHO (29). In the US, in observation of FDA recommendations, blood collection was halted in Puerto Rico in between March and April 2016, resumed when NAT screening was introduced in late April. Approximately 0.5% of the donors were found viremic, peaking to 1.8% in July 2016 (30). Mosquito-borne and travel-associated cases in the continental US led the FDA to extend the recommendation of NAT screening to the whole country, being implemented by September/October 2016 and indeed depicting some infected donors, the majority with a recent travel history to an endemic area (31, 32). No country in LATAM so far followed this policy. The sharp decline in Zika incidence in LATAM in 2017 reduced the pressure and debate over this issue. High seroprevalence rates are observed today in the most affected areas of Brazil, what may prevent new large outbreaks in the near future (22, 33).

### Chikungunya

Chikungunya (CHKV), in common with Zika and Dengue, is also transmitted by mosquitos from the *Aedes* genus, causing similar symptoms, making difficult to perform a diagnosis relying solely on clinical manifestations. Noticeably, arthralgia is much more pronounced and may last for months in some patients.

Its transmission by blood transfusion remains theoretical since no single case of TT-CHKV has ever been published. CHKV was introduced to LATAM in 2013, hitting first the Saint Martin Island in the Caribbean, brought probably from the South Pacific. The implicated CHKV Asian strain rapidly spread over the Caribbean and to South, Central, and North America (34). In the French West Indies, concerns about blood safety led to early implementation of a lab-developed NAT, in addition to pathogen reduction and quarantine (35). They were able to detect four viremic donors, two of them developed fever after donation and the other two remained asymptomatic. A large outbreak occurred a few months later in Puerto Rico with up to 2.1% of donors testing CHKV-RNA^+^ (36). In contrast to the fast adoption of NAT for WNV, upon solid evidence of the clinical impact of TT-WNV, and for Zika, even lacking such parallel data, NAT for CHKV was never implemented in Puerto Rico.

From the Caribbean, the Asian strain spread first to the Northern countries of South America; Colombia, Venezuela, Suriname, Guyana, and the French overseas territory of French Guyana (37). The Brazilian Amazon state of Amapá, contiguous to the French Guyana, was the first to report autochthonous cases in September 2014. Curiously, at about the same time, an infected individual brought the East–South–Central Africa (ECSA) strain to the Northeastern state of Bahia, resulting in hundreds of cases and the establishment of this lineage as endemic in the region (38). In the following years, growing number of presumed CHKV cases were verified in Brazil, 38,499 in 2015, 271,824 in 2016, and approximately 200,000 in 2017, with dozens of deaths (39, 40). It is suspected that another arbovirus, Mayaro (MAYV), belonging to the same Alphavirus genus from the *Togaviridae* family, may be hidden among cases attributed to CHKV (41) and DENV (42).

There is a fear in LATAM in general, that huge outbreaks of CHKV will take place in the next years, since the majority of the population is still naïve to this virus. Strategies to mitigate the risk of TT-CHKV are not being actively discussed, since the risk of getting infected by mosquitoes is much higher and the associated clinical picture absolutely clear. In LATAM countries, with several social and health demands, it is debatable whether the resources to prevent a few TT-CHKV should not be invested in vector control, in order to benefit a larger number of inhabitants, including blood recipients that are off course also susceptible to mosquitoes’ bites in daily life outside blood transfusion settings. However, availability of, in development, arboviral multiplex NATs allowing for simultaneous detection of Dengue, Chikungunya, and Zika and/or pathogen reduction technologies acting on whole blood (43) or components, necessarily including red cells (44) may perhaps result in cost-effective measures to be implemented in endemic areas, home to the majority of the LATAM population.

## Author Contributions

The author confirms being the sole contributor of this work and approved it for publication.

## Conflict of Interest Statement

The author declares that the research was conducted in the absence of any commercial or financial relationships that could be construed as a potential conflict of interest.
